# A linear time algorithm for detecting long genomic regions enriched with a specific combination of epigenetic states

**DOI:** 10.1186/1471-2164-16-S2-S8

**Published:** 2015-01-21

**Authors:** Kazuki Ichikawa, Shinichi Morishita

**Affiliations:** 1 Department of Computational Biology, Graduate School of Frontier Sciences, The University of Tokyo, Kashiwa 277-0882, Japan

## Abstract

**Background:**

Epigenetic modifications are essential for controlling gene expression. Recent studies have shown that not only single epigenetic modifications but also combinations of multiple epigenetic modifications play vital roles in gene regulation. A striking example is the long hypomethylated regions enriched with modified H3K27me3 (called, "K27HMD" regions), which are exposed to suppress the expression of key developmental genes relevant to cellular development and differentiation during embryonic stages in vertebrates. It is thus a biologically important issue to develop an effective optimization algorithm for detecting long DNA regions (*e.g*., >4 kbp in size) that harbor a specific combination of epigenetic modifications (*e.g*., K27HMD regions). However, to date, optimization algorithms for these purposes have received little attention, and available methods are still heuristic and *ad hoc*.

**Results:**

In this paper, we propose a linear time algorithm for calculating a set of non-overlapping regions that maximizes the sum of similarities between the vector of focal epigenetic states and the vectors of raw epigenetic states at DNA positions in the set of regions. The average elapsed time to process the epigenetic data of any of human chromosomes was less than 2 seconds on an Intel Xeon CPU. To demonstrate the effectiveness of the algorithm, we estimated large K27HMD regions in the medaka and human genomes using our method, ChromHMM, and a heuristic method.

**Conclusions:**

We confirmed that the advantages of our method over those of the two other methods. Our method is flexible enough to handle other types of epigenetic combinations. The program that implements the method is called "CSMinfinder" and is made available at: http://mlab.cb.k.u-tokyo.ac.jp/~ichikawa/Segmentation/

## Background

Epigenetic modifications have been shown to play a vital role in regulating gene expression. Recent genome-wide studies have revealed that in vertebrates, although most CpG sites in DNA sequences are highly methylated, hypomethylated CpG islands proximal to genes are involved in regulating gene expression [1]. Specifically, hypermethylated CpG islands in promoter regions are relevant to gene silencing, while hypomethylated CpG islands are in an active or permissive state for transcription [2]. In addition to cytosine methylation of CpG sites, some histone modifications around promoter regions also are known to affect the regulation of gene expression [3,4].

**Figure 1:**
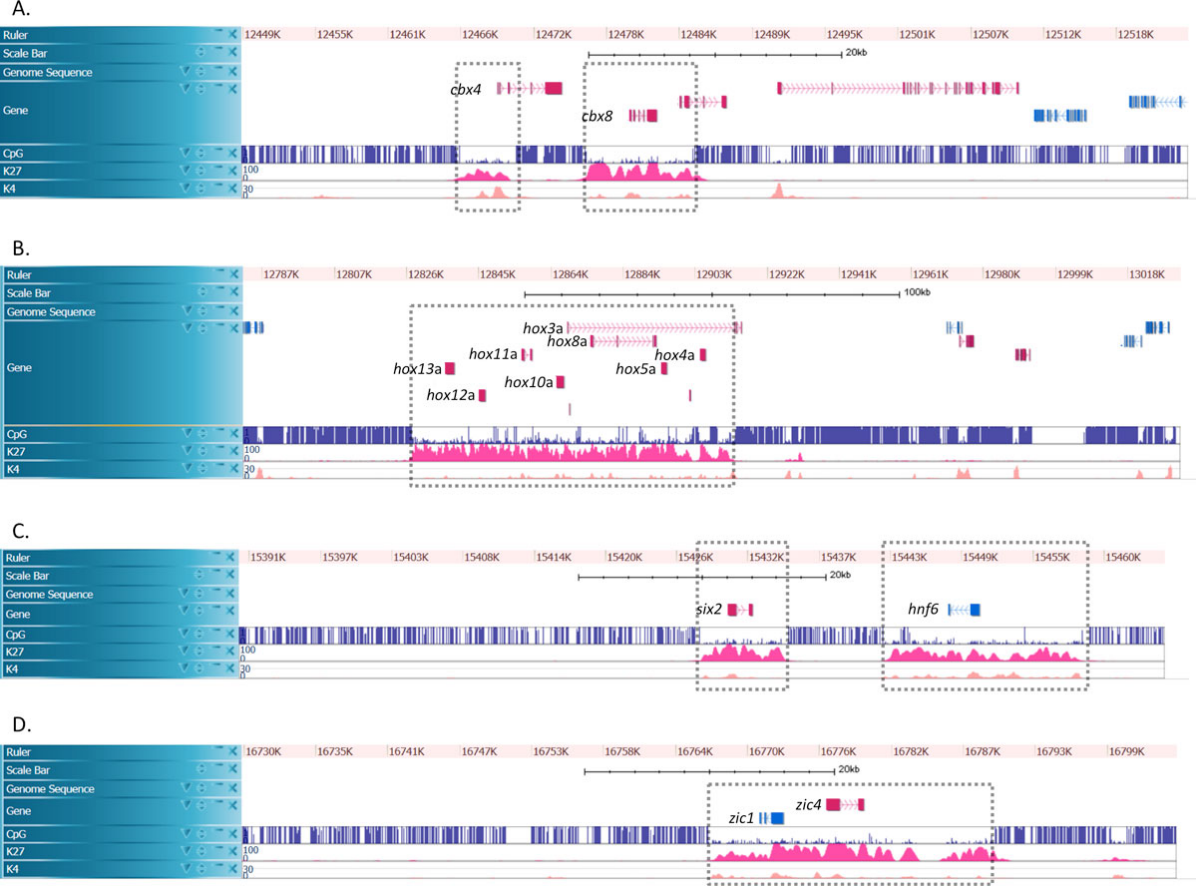
**Examples of long K27HMD regions in the medaka genome**. Examples of K27HMD regions enclosed in dashed boxes. Each screen capture shows an image in a medaka genome browser that displays tracks of gene structures, CpG methylation levels observed by bisulfite sequencing, and levels of H3K27me3 and H3K4me2 in blastula cells (half-day embryos). **A**. A K27HMD region of length ~4 kbp with *cbx4*, and a ~8 kbp region with *cbx8*. **B**. A large region of length ~90 kbp with *hoxa *genes. **C**. A ~6 kbp region with *six2*, and a ~14 kbp region with *hnf6*. **D**. A ~20 kbp region with *zic1 *and *zic4*.

It was found recently that long hypomethylated regions enriched with H3K27me3 were likely to overlap with regions encoding key genes essential for cell development and differentiation in human embryonic stem cells [5], mouse hematopoietic stem cells [6], early *Xenopus tropicalis *embryos demonstrates [7], and medaka fish blastula (half-day) embryos [8]. Although many hypomethylated domains (HMD) are subjected to modification of the active histone mark H3K4me2 that promotes gene expression [9-12], it is remarkable that ~300 HMDs of length >4 kb rarely have H3K4me2 histone marks but have repressive H3K27me3 histone marks, and are found in association mostly with developmental genes [8]. Promoters in HMD with H3K27me3 marks (called, "K27HMD") are in a 'poised' state, in which the genes are not simply silenced but are ready for activation immediately during cell differentiation, which is important for sustaining the pluripotency of pluripotent cells [13,14]. Figure 1 shows four examples of long K27HMD regions that include developmental genes such as *cbx4*, *cbx8*, *hoxa *genes, *six2*, *hnf6*, and *zic1/4*.

Thus, there has been considerable interest in long K27HMD regions with biologically important characteristics. However, computational methods for detecting long K27HMD regions are still heuristic and *ad hoc*, emphasizing the need to develop an effective algorithm from a profound background in computation theory. For example, to identify K27HMD, Nakamura et al. proposed a heuristic method that used certain *ad hoc *parameter settings to define hypomethylated regions and H3K27me3 peak detection [8]. The method is not guaranteed to output K27HMD regions longer than a given threshold, and it often generates regions of differing lengths. ChromHMM [15] is a statistical method that classifies epigenetic modifications into classes of combinations and divides a DNA sequence into sub-regions such that each sub-region has a uniform combination of epigenetic states while neighboring sub-regions have distinct characteristics. ChromHMM has been used successfully to partition regions surrounding genes into active/inactive promoters, exons, and introns by analyzing epigenetic codes. Although ChromHMM can be used for K27HMD detection by setting its parameters to find regions that are hypomethylated and marked by H3K27me3, ChromHMM often generates many short regions and thus is not suitable for detecting large K27HMD regions. Overall, these previous methods have simply not been designed to output regions of lengths greater than or equal to a given minimum threshold.

To address this problem, we propose a linear time algorithm for calculating a set of non-overlapping regions such that the set maximizes the score of focal combinations of epigenetic modifications (*e.g*., K27HMD) and the length of each region is greater than or equal to a given minimum threshold (*e.g*., 4 kb). We define the score of a focal combination of epigenetic modifications at each DNA position as the similarity between the vector of focal epigenetic states and the vector of raw epigenetic states at the position. We then define the similarity score of a set of regions as the sum of similarity scores of all positions in the set. This method solves several issues in previous heuristic methods because it allows us to set a minimum region length for detecting 'long' regions of biological importance and guarantees the output of an optimal set of long regions that maximizes an objective function.

We implemented the algorithm. We call the program CSMinfinder (Chromatin State with minimum length finder). With CSMinfinder, we identified large K27HMD regions in the medaka and human genomes [8,16,17] that overlapped many developmental genes. CSMinfinder can be applied to epigenetic data from other vertebrates for understanding cell development and differentiation.

CSMinfinder runs in time proportional to the size of the genome, and it can process vertebrate genomes in feasible amounts of time. Although we applied CSMinfinder specifically to K27HMD, it can be used for the detection of regions with other types of epigenetic combinations by defining the vector of focal epigenetic states appropriately.

## Methods

To detect long regions of focal epigenetic states, we formulated this as a problem of finding an optimal set of disjoint (non-overlapping) regions in a sequence that maximizes the sum of similarity scores in all regions. Our method calculates a similarity score between a vector of epigenetic modifications at each position and the feature vector of a focal epigenetic state, such as K27HMD, and outputs the set of regions with the highest sum of similarity scores.

### Calculating a similarity vector

We need to generate a modification vector at each position from epigenomic signal data. For example, to create benchmark datasets in this study, we binarized the modification signal level at each position using BinarizeBed in ChromHMM [15], which classified the signal at each position into 0 or 1 according to a Poisson background model. Subsequently, we defined a modification vector as the vector with binary scores of modifications at each position.

**Definition 1**. Let *w*_l_,*w*_2_...,*w_n _*be non-overlapping windows of the same length (e.g., 200 bp in this study) in a DNA sequence. Let $s_{i}^{1},\ldots ,s_{i}^{k}$ be binary or real-valued signals of *k *modifications in window *i*. The modification vector of *w_i _*is defined as $M_{i}=\left(s_{i}^{1},\ldots ,s_{i}^{k}\right)$. Let *F *denote the feature vector of a focal modification pattern with *k *elements. The similarity score of *M_i _*and *F *is defined as their inner product minus a given threshold *τ*.

**Example**. Suppose that *k *= 3, *τ *= 1.3, *F *= (1,1,0), *M*_1 _= (1,1,0), *M*_2 _= (1,0,1) and *M*_3 _= (0,0,1). Similarity scores of *F *and *M_i _*are 0.7, -0.3, and -1.3 for *i *= 1,2,3

When the inner product of *M_i _*and *F *is positive for all *i *= 1,...,*n*, the optimal set of regions that maximizes the sum of similarity scores in the regions becomes the entire region, [1,*n*], which may not be informative. If we want to select a set of regions whose modification vectors are closer to the feature vector *F*, we can set the threshold *τ *to an appropriate positive value to yield a negative similarity score for the inner product that is lower than *τ*. Positions with negative similarity scores are less likely to be included in the optimal set of regions. A higher threshold is likely to divide the entire genome into smaller regions with a higher precision, while a lower threshold yields an opposite trend. In this manner, for a series of windows *w*_l_,*w*_2_...,*w_n _*in a DNA sequence, we generate a series of similarity scores.

### Detecting an optimal set of disjoint regions

To detect regions of focal epigenetic states such as K27HMD, we present an algorithm for calculating an optimal set of disjoint regions in a sequence that maximizes the sum of similarity scores for all regions. In addition, to identify sufficiently long regions, we define a minimum length threshold of regions such that each region is longer than or equal to the minimum length. The problem can be defined as follows.

**Definition 2**. Let *L *= {*L_i_*|*i *= 1,2,...,*n*} be a series of real valued weights *L_i _*(*e.g*., similarity scores). Let *C *be a series of non-overlapping regions *I_j _*(*j *= 1,...*k*) of *L *such that the length of each *I_j _*is greater than or equal to a given minimum threshold *m*_1_, and the length of the interval between *I*_*j*-1 _and *I*_*j *_is greater than or equal to another given minimum threshold *m*_0_. That is, *C *is a series of regions of the form $\left\{\left[a_{1},b_{1}\right],\cdots \left[a_{k},b_{k}\right]\right\}\left(1\leq a_{1}<b_{1}<a_{2}<b_{2}\cdots <a_{k}<b_{k}\leq \text{n}\right)$ such that

1. *a_t _*+ *m*_1 _- 1 ≤ *b_t _*for *t *= 1,...,*k *(the minimum length constraint on regions),

2. *b*_*t*-1_+*m*_0 _<*a_t _*for *t *= 2,...,*k *(the minimum length constraint on intervals between regions), and

3. *a*_1 _= 1 or *a*_1 _ >*m*_0 _(the first region start at position 1 or at position larger than *m*_0_).

Readers may find the last condition strange because it appears to disallow the situation that the first region starts at position *a*_1 _>*m*_0_. We used the condition to simplify the presentation of our linear-time algorithm, which is described later. To obtain such an optimal series of regions that the first region starts at *a*_1 _>*m*_0_, for example, you can temporarily add *m*_0 _negative weights in front of *L*, calculate the optimal series, and restore the coordinate.

To calculate a *C *that maximizes the sum of weights in *C*, $\underset{i\in I\in C}{\sum }L_{i}$, we used a dynamic programming algorithm developed by Csuros [18]. Here, we outline the algorithm.

**Definition 3**. We assume that all series meet the conditions given in Definition 2. Let *w*(*C*) denote the sum of weights in *C*, $\underset{i\in I\in C}{\sum }L_{i}$. We consider two cases: that in which the last region of *C *ends at *I *and that in which it does not. When the last region does not end at *i*, let $C_{i,m}^{0}$ denote a series of regions that maximizes *w*(*C*) among all series, such that the last region ends at position *b_k _*≤ *i *- *m*, where *m *≥ 1. When the last region ends at *i*, let $C_{i,m}^{1}$ denote a series of regions that maximizes *w*(*C*) among all series, such that the last region is of length ≥ *m *(≥1); specifically, *a_k _*+ *m *- 1 ≤ *i *(= *b_k_*).

**Example**. When *i *= 12, and *L *= (1,1,-3,1,1,-3,1,1,1,1,-2,1), we have

$$\begin{array}{c} C_{12,1}^{0}=\left\{\left[1,2\right],\left[4.5\right],\left[7,10\right]\right\},\;C_{12,4}^{0}=\left\{\left[1,2\right],\left[4,5\right],\left[7,8\right]\right\},\\ C_{12,7}^{1}=\left\{\left[1,2\right],\left[4,12\right]\right\},\;C_{12,12}^{1}=\left\{\left[1,12\right]\right\}. \end{array}$$

According to this definition, *C *maximizing *w*(*C*) is either $C_{n,1}^{0}$ or $C_{n,m_{1}}^{1}$. For calculating these two series, we define here $w\left(C_{i,m}^{0}\right)$ and $w\left(C_{i,m}^{1}\right)$ recursively for *i *= 1,...,*n *and *m *≥ 1.

**Definition 4**. We define the following four types of weight sums, $W_{short}^{0}\left(i\right)$, $W_{long}^{0}\left(i\right)$, $W_{short}^{1}\left(i\right)$, and $W_{long}^{1}\left(i\right)$, depending on whether the last region ends at *i *or not (denoted as 1 or 0, respectively) and whether the minimum length constraint is satisfied or not (denoted as *long *or *short*, respectively):

$$\begin{array}{c} W_{short}^{0}\left(1\right)=0,\;W_{short}^{1}\left(1\right)=L_{1},\\ W_{short}^{0}\left(i\right)=w\left(C_{i,1}^{0}\right),\;W_{long}^{0}\left(i\right)=w\left(C_{i,m_{0}}^{0}\right),\\ W_{short}^{1}\left(i\right)=w\left(C_{i,1}^{1}\right),\;W_{long}^{1}\left(i\right)=w\left(C_{i,m_{1}}^{1}\right) \end{array}$$

Csuros showed that these four types of weight sums can be calculated recursively as follows [18]:

$$\begin{array}{c} W_{short}^{0}\left(i\right)=max\left\{W_{short}^{0}\left(i-1\right),W_{long}^{1}\left(i-1\right)\right\}\;\text{for}\;i\in \left[2,n\right]\\ W_{short}^{1}\left(i\right)=L_{i}+max\left\{W_{long}^{0}\left(i-1\right),W_{short}^{1}\left(i-1\right)\right\}\;\text{for}\;i\in \left[2,n\right]\\ W_{long}^{0}\left(i\right)=W_{short}^{0}\left(i-m_{0}+1\right)\;\text{for}\;i\in \left[m_{0},n\right]\\ W_{long}^{1}\left(i\right)=W_{short}^{1}\left(i-m_{1}-1\right)+\overset{i}{\underset{j=i-m_{1}+2}{\sum }}L_{j}\;\text{for}\;i\in \left[m_{1},n\right] \end{array}$$

Recall that *C *maximizing *w*(*C*) is either $C_{n,1}^{0}$ or $C_{n,m_{1}}^{1}$. From $W_{long}^{1}\left(n\right)$, we can build the series of regions, $C_{n,m_{1}}^{1}$, by tracing back the process of calculating $W_{long}^{1}\left(n\right)$. Similarly, from $W_{short}^{0}\left(n\right)$, we can obtain $C_{n,1}^{0}$.

We implemented the above idea. We call the program CSMinfinder.

## Results

### Data sets

To compare CSMinfinder with other available methods for detecting large K27HMD, we used real biological datasets from the medaka-fish and human genomes, each of which was a set of vectors of DNA methylation levels at CpG sites, determined by bisulfite sequencing, and H3K4me3 and H3K27me3 histone modification Chip-seq data [8]. We set the window size to 200 bp, normalized the data using a Poisson distribution model, and set the binarized score of a window to 1 if its probability was < 0.0001 and to 0 otherwise.

### Detecting large K27HMD in medaka epigenomic data

We compared CSMinfinder with ChromHMM [15] and Nakamura's method [8].

• Using ChromHMM, we estimated six chromatin states and divided the given DNA sequence into these six states. Specifically, ChromHMM asks users to input the number of epigenetic states beforehand. Thus, we started with inputting a small number into ChromHMM, increased the number gradually one by one until we found a state similar to K27HMD, hypo-methylated DNA modification and H3K27me3 histone modification, and called the number *sufficient*. Inputting a value larger than the sufficient number into ChromHMM did not make much sense because it just output a state similar to K27HMD. The sufficient number was six. Among the six states, one represented hypomethylated DNA modifications and the H3K27me3 histone modification. We therefore treated the state as K27HMD.

• Nakamura's method detects a hypomethylated domain on a DNA sequence that has more than nine contiguous CpG sites with low methylation (methylation level <0.4) and no more than four contiguous highly methylated CpG sites. Parameters are selected heuristically. A hypomethylated domain is treated as a K27HMD if it contains H3K27me3 peaks detected by QuEST [19], such that each peak is more than three times larger than the average.

• In CSMinfinder, we used two types of minimum length thresholds, 4 kbp and 8 kbp, to evaluate the effect of this constraint. We set the minimum length of any interval between regions to 600 bp.

### Comparing the performance in detection of large K27HMD around genes in the medaka genome

Large K27HMD regions of length >4 kbp suppress the expression of many developmental genes [8]. Thus, we verified the effectiveness of CSMinfinder for detecting large K27HMD regions surrounding genes in the medaka genome. Nakamura's method could detect 246 large K27HMD regions containing the promoter regions of developmental genes (e.g., *hox *clusters) that were relevant to transcriptional regulation and the developmental process. CSMinfinder detected 911 K27HMD regions, and of these, 386 regions contained promoter regions of >4 kbp in size and contained 242 of the 246 regions identified using Nakamura's method. Indeed, our regions covered 91% of bases in the entire regions detected by Nakamura's method. Specifically, although the exact boundaries of individual regions estimated by the two methods were unlikely to be consistent, these regions largely overlapped each other. These results demonstrate the high concordance between CSMinfinder and Nakamura's methods as well as the ability of CSMinfinder to identify more K27HMD regions than did Nakamura's method.

**Figure 2:**
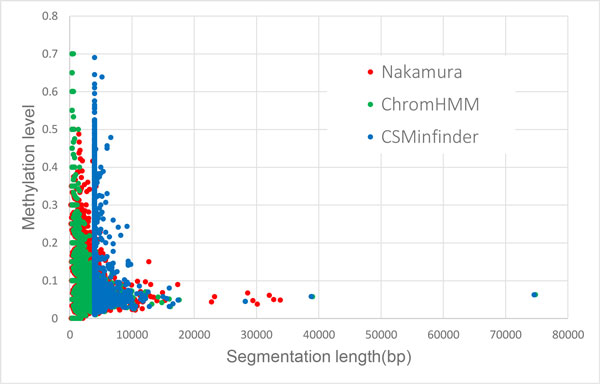
**Lengths and average methylation levels of K27HMD regions in the medaka genome**. Each dot represents a region that is identified by CSMinfinder, ChromHMM, and Nakamura's method in the medaka genome. The *x*-axis shows the length of a K27HMD region and the *y*-axis presents the average methylation level of the region.

We assessed the quality of each K27HMD region in terms of their low average DNA methylation level because this property is considered to be essential in maintaining the suppression of developmental gene expression in embryonic cells [8]. Indeed, Figure 2 shows the tendency of the average methylation level in the vertical axis to become lower for a longer K27HDM region, the length of which is displayed in the horizontal axis. This trend was also observed with all three methods.

**Figure 3:**
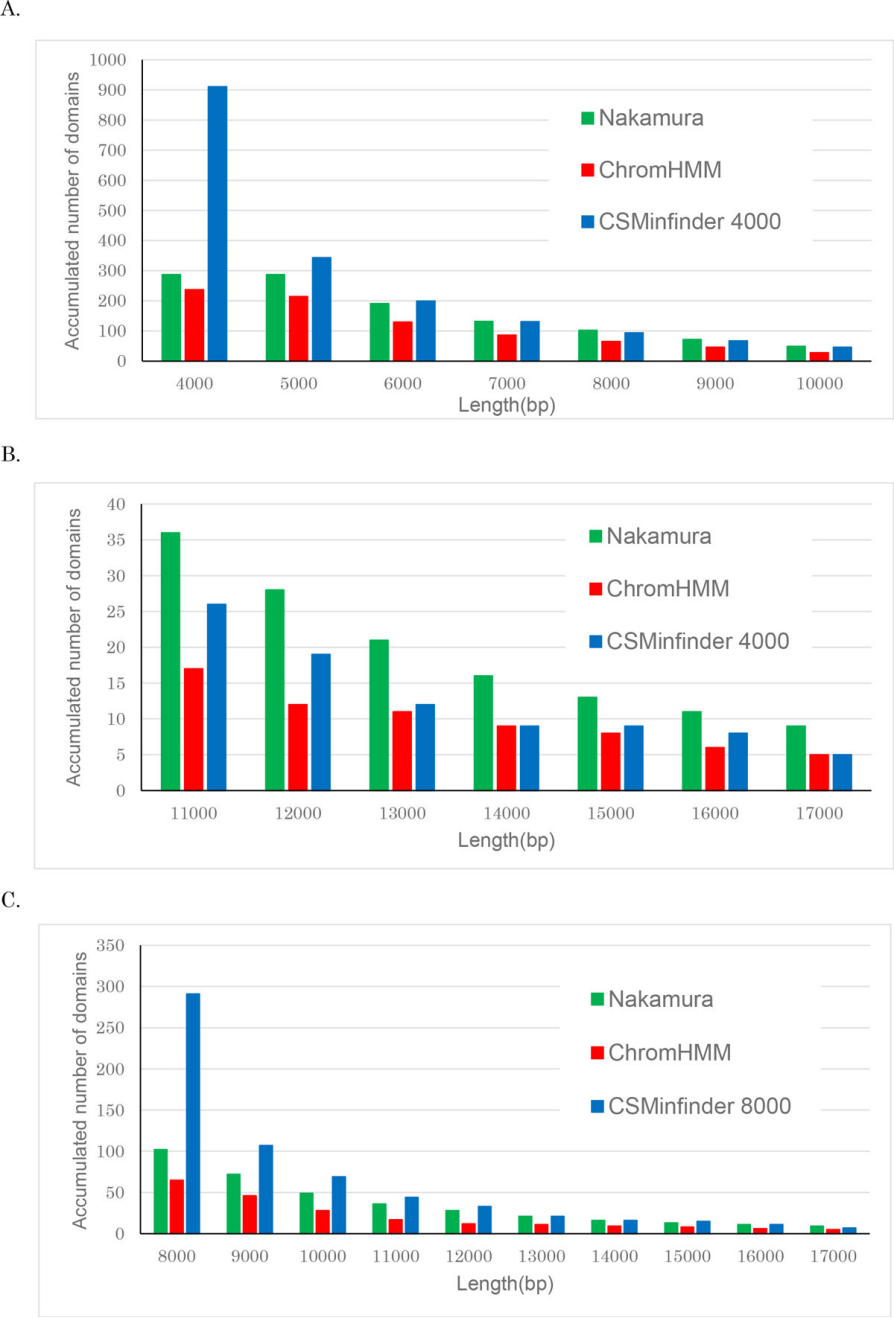
**Length distribution of large K27HMD regions in the medaka genome**. **A-B**. Comparison between CSMinfinder (minimum length threshold of 4 kbp), ChromHMM, and Nakamura's method. The *x*-axis shows the minimum length of K27HMD regions, and the *y*-axis shows the accumulated number of K27HMD regions longer than or equal to the threshold in the *x*-axis. Because of the space limitations, the histogram is divided into two sub-histograms **A **(threshold is ≤ 10 kbp) and **B **(threshold ≥ 11 kbp). **C**. In this case, we set the minimum threshold to 8 kbp using CSMinfinder.

We then compared the performance of the three methods by examining the length distributions of K27HMD regions in the medaka genome. Figure 3A shows the length distributions of large K27HMD regions (>4 kb in size) estimated by each of the three methods. Setting the minimum length threshold to 4 kbp in CSMinfinder detected more regions of length > 6 kbp but fewer regions of length >7 kbp compared with Nakamura's method. CSMinfinder allows us to output longer regions by setting the minimum length threshold to a higher value. For example, setting the minimum length to 8 kbp, CSMinfinder found more regions than did Nakamura's method (Figure 3C).

### Analysis of large K27HMD regions in human epigenomic data

We also compared CSMinfinder with the other two for processing human epigenomic data. For ChromHMM, we calculated the sufficient number for the human data according to the procedure described before, and we classified epigenetic modification data into seven states rather than six so as to identify a state similar to K27HMD. The sufficient numbers of epigenetic states in the human and medaka data differed due to the difference in data quality. The sufficient number in the medaka data was smaller than that in the human data presumably because epigenetic state signals in the medaka data were clearer.

In CSMinfinder, we set the minimum length threshold to 8 kbp and the interval between regions to 600 bp. We also searched an ideal value of threshold τ by repeated trials to detect large continual regions, and we set τ to 1.3 and 1.6 in the respective medaka and human data.

**Figure 4:**
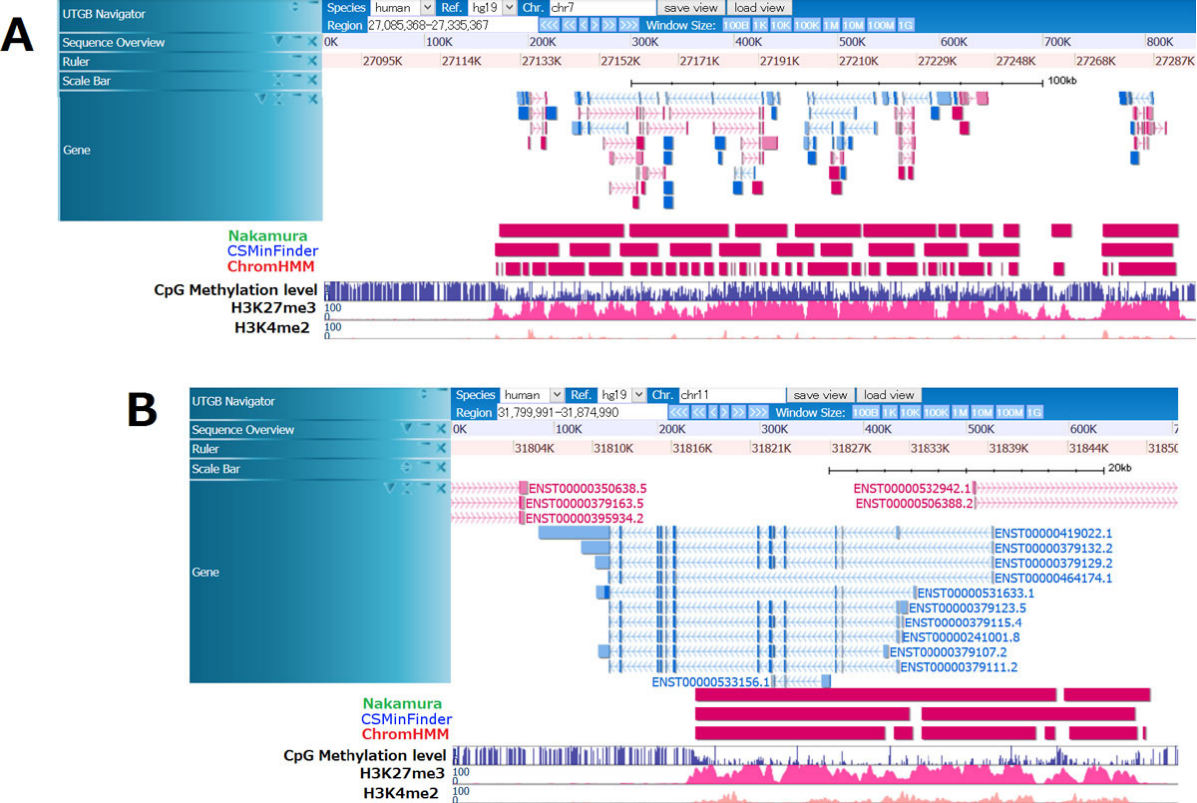
**Examples of large K27HMD regions around developmental genes in the human genome**. **A**. The figure displays several K27HMD regions in the human chromosome 11 around *pax6*, a gene that regulates eye and brain development. CSMinfinder and Nakamura's method detected large K27HMD regions of >4 kbp in size and output large regions that largely overlapped; however, ChromHMM divided these regions into smaller ones. **B**. These large K27HMD on human chromosome 7 were located around a cluster of *hox *genes that regulate the body plan of the head-tail axis. ChromHMM yielded much smaller K27HMD regions as output than did the other two methods.

**Figure 5:**
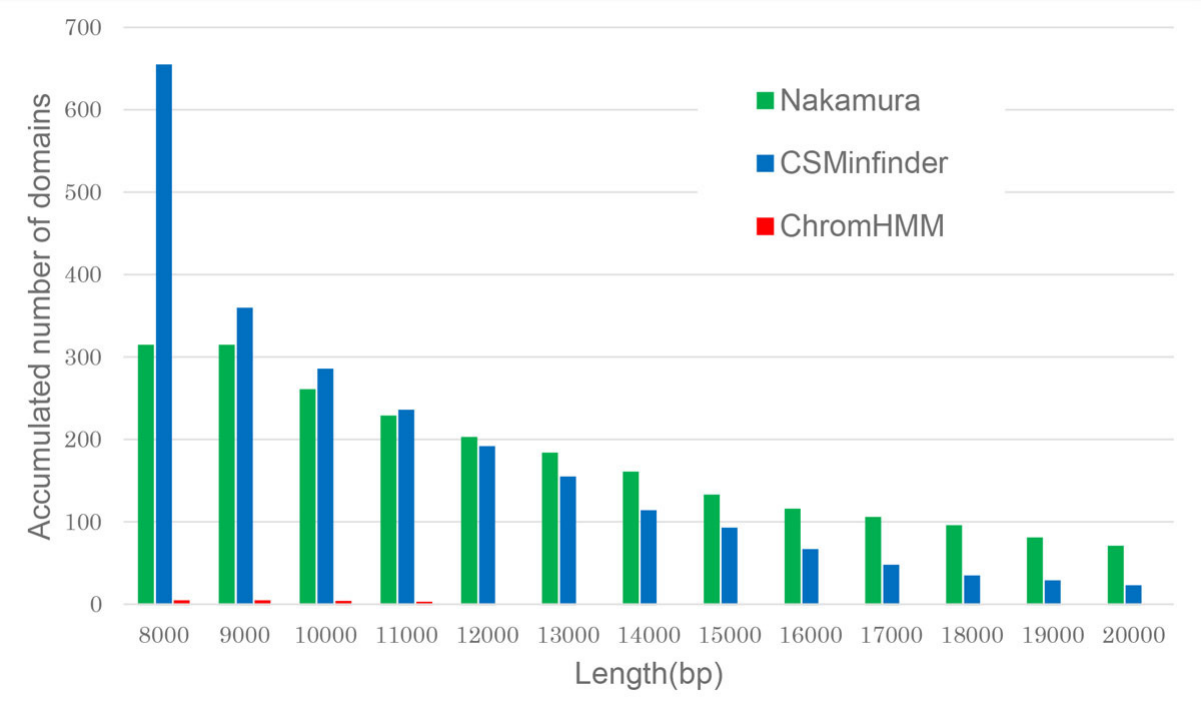
**Length distribution of large K27HMD regions in the human genome**. Comparison between CSMinfinder (minimum length threshold of 8 kbp), ChromHMM, and Nakamura's method. The *x*-axis shows the minimum K27HMD region length threshold, and the *y*-axis shows the accumulated number of K27HMD regions longer than or equal to the threshold on the x-axis.

Because the human genome is longer than the medaka genome, we focused on large K27HMD regions of length > 8 kbp. Nakamura's method detected 314 regions, and CSMinfinder identified 542 regions, including 291 of those found using Nakamura's method. Again, there was high concordance between the results obtained by the two methods. Figure 4 shows examples of large K27HMD regions detected around developmental genes. Although CSMinfinder and Nakamura's method yielded slightly different regions with distinct boundaries in the output, each created regions of similar sizes. In contrast, ChromHMM yielded shorter regions than the other two did. Specifically we compared the length distribution of large K27HMDregions estimated by each of the three methods (Figure 5). We found that CSMinfinder and Nakamura's method were comparable. Precisely, although the number of extremely large regions longer than 12 kbp is slightly smaller than the number found by Nakamura's method, CSMinfinder could detect similar numbers of large regions between 8 kbp to 12 kbp. Later we will discuss the reason why ChromHMM were inferior to the other two methods.

### Computational performance and software availability

**Figure 6:**
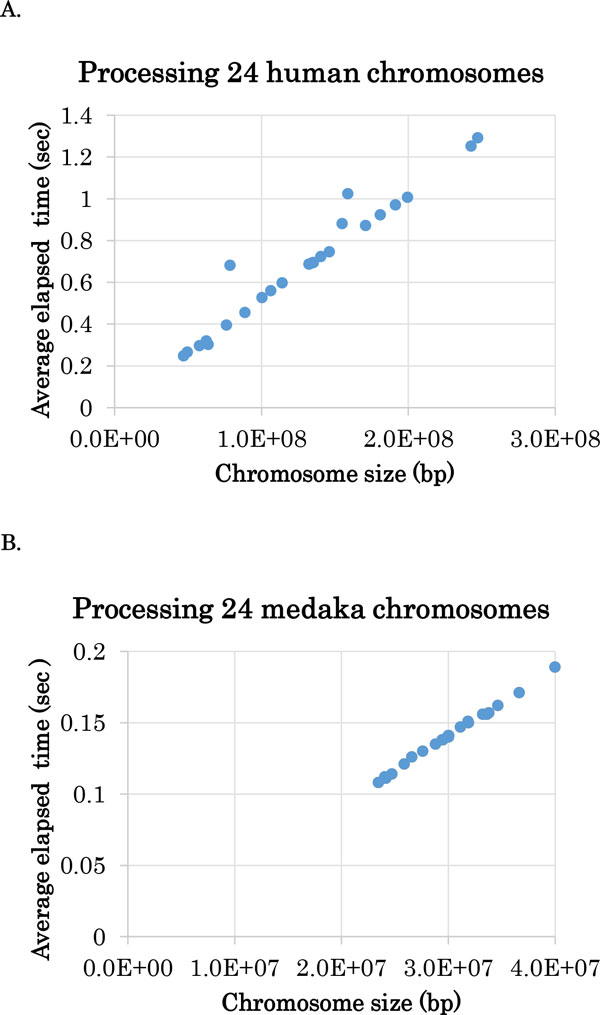
**Average elapsed time of processing human (A) and medaka (B) chromosomes ten times by using CSMinfinder**. The minimum threshold is set to 8 kbp for handing the human genome, and 4 kbp for the medaka genome. Each dot represents a chromosome, the x-axis value shows the size of the chromosome, and the y-axis value is the average elapsed time.

We observed the computational performance of CSMinfinder using Intel Xeon CPU E5-2670 processor with a clock rate of 2.60 GHz and 66GB of main memory. The computation time to calculate the optimal series of regions was negligible. Figure 6 shows that the average elapsed time was less than 2 seconds when we processed the epigenetic data of any of human and medaka chromosomes. Furthermore, Figure 6 also illustrates that the elapsed time is almost proportional to the size of each chromosome, thereby confirming experimentally that the worst-case time complexity of the algorithm is linear in the input size. CSMinfinder does not consume a large amount of main memory. CSMinfinder is made available at the following site: URL: http://mlab.cb.k.u-tokyo.ac.jp/~ichikawa/Segmentation/

## Conclusions and discussion

In this work, we proposed a method that estimates large K27HMD regions [5-8,12] by calculating the similarity between the vector of focal epigenetic states and that of raw epigenetic states at each DNA position. The advantage of this algorithm (CSMinfinder) is the output of an optimal series of regions while allowing us to set the minimum length threshold on individual regions. We estimated large K27HMD in the medaka and human genomes and verified that CSMinfinder was comparable to Nakamura's heuristic method [8] designed to detect K27HMD and was advantageous over ChromHMM in terms of the lengths of K27HMD regions.

For the medaka genomic data, ChromHMM performed well and could detect as many long regions as CSMinfinder did; however, for the human genomic data, ChoromHMM found a smaller number of large K27HMD regions of length > 8 kbp than the other two methods did. This was likely due to the differences in characteristics between the medaka and human genomic data. In the medaka genome, the data were collected from an inbred stain in which the genomic differences between the two alleles were quite small. Thus, methylation levels were bimodal and were clearly divided into two states, hypomethylated and hypermethylated, making it relatively easy to identify blocks of hypomethylated domains. In the human genome, however, the majority of methylation levels were poised because the human genome is diploid intrinsically and allele-specific methylation is prevalent, making it more difficult to detect clear boundaries between hypermethylated and hypomethylated domains. Although many DNA methylation levels are ambiguous in the human genome, ChromHMM attempts to assign one state to each position. Positions with vague DNA methylation levels are assigned only a single state by ChromHMM. Thus, ChromHMM is likely to output too many short regions.

One advantage of CSMinfinder is that we can set the minimum region length for specific purposes. For example, in the medaka genome, using an 8-kbp minimum length threshold merged some of the shorter regions that were generated using a 4-kbp minimum threshold into a longer continuous region. Thus, we could obtain longer regions using a higher minimum length threshold. Similarly, we can also adjust the minimum threshold for defining similarity scores between modification vectors and the feature vector for a variety of purposes. Setting the minimum threshold to a lower value generates more regions that are less similar to the feature vector of interest. Having more than one series of regions that may overlap can be informative. We can therefore tune CSMinfinder easily to meet various demands.

In this paper, we demonstrated the advantages of our algorithm by detecting large K27HMD regions that have attracted much interest because of their importance in characterizing the behavior of developmental genes and confirmed the performance of our algorithm. CSMinfinder is not limited to the identification of large K27HMD regions but can be used for the detection of other large DNA regions that have different types of epigenetic state combinations associated with regulating gene functions.

## Competing interests

The authors declare that they have no competing interests.
